# Pooled prevalence and risk factors of malaria among children aged 6–59 months in 13 sub-Saharan African countries: A multilevel analysis using recent malaria indicator surveys

**DOI:** 10.1371/journal.pone.0285265

**Published:** 2023-05-31

**Authors:** Dagmawi Chilot, Annelies Mondelaers, Adugnaw Zeleke Alem, Mezgebu Selamsew Asres, Mulugeta Ayalew Yimer, Alemayehu Teklu Toni, Tadesse Awoke Ayele

**Affiliations:** 1 Department of Human Physiology, University of Gondar, College of Medicine and Health Science, School of Medicine, Gondar, Ethiopia; 2 Department of Clinical Sciences, Institute of Tropical Medicine, Antwerp, Belgium; 3 Department of Epidemiology & Biostatistics, Institute of Public Health, University of Gondar, College of Medicine and Health Science, Gondar, Ethiopia; 4 Department of Internal Medicine, College of Medicine and Health Sciences, University of Gondar, Gondar, Ethiopia; 5 Department of Pediatrics and Child Health, College of Medicine and Health Sciences, University of Gondar, Gondar, Ethiopia; Hawassa University College of Medicine and Health Sciences, ETHIOPIA

## Abstract

**Background:**

Every 75 seconds, a child under five dies of malaria. Mainly children, aged between six months and five years, are at the highest risk for malaria. These children lost maternal immunity and did not yet developed specific immunity to the infection. Under the age of five, children bear the highest burden of malaria in Sub-Saharan Africa (SSA). Many individual and community level factors could contribute to malaria prevalence remaining high among under-five children in the region. Thus, this study aimed to assess the pooled prevalence of malaria among children aged 6–59 months and identify potential factors associated with malaria by using recent Malaria Indicator Surveys in 13 SSA countries.

**Methods:**

Data for this study were drawn from recent 13 Sub-Saharan African countries Malaria Indicator Surveys (MIS). A total weighted sample of 60,541 children aged 6–59 months was included. STATA version 14.2 was used to clean, code and analyze the data. Multilevel logistic regression was employed to identify factors associated with malaria. Adjusted odds ratio with 95% CI and a P value <0.05 was reported to indicate statistical association. Model fitness and comparison were done using Inter cluster correlation coefficient, Median odds ratio, proportional change in variance, and deviance.

**Results:**

The pooled prevalence of malaria among children aged 6–59 months was found to be 27.41% (95% CI: 17.94%-36.88%). It ranges from 5.04% in Senegal to 62.57% in Sierra Leone. Aged 36–47 months (AOR = 3.54, 95% CI 3.21–3.91), and 48–59 months (AOR = 4.32, 95% CI 3.91–4.77), mothers attended primary education (AOR = 0.78, 95% CI 0.73–0.84), richer (AOR = 0.35, 95% CI 0.32–0.39), and richest household (AOR = 0.16, 95% CI 0.14–0.19), number of three and more under-five children (AOR = 1.35, 95% CI 1.26–1.45), improved floor material (AOR = 0.65, 95% CI 0.57–0.73), improved wall material (AOR = 0.73, 95% CI 0.64–0.84), improved roof material (AOR = 0.70, 95% CI 0.51–0.93), insecticide-treated bed net (ITN) use (0.56, 95% CI 0.51–0.62), not anemic (AOR = 0.05, 95% CI 0.04–0.06), rural resident (AOR = 2.16, 95% CI 2.06–2.27), high community ITN use (AOR = 0.40, 95% CI 0.24–0.63) and high community poverty (AOR = 2.66, 95% CI 2.53–2.84) were strongly associated with malaria.

**Conclusions and recommendations:**

Almost 3 out of 10 children were infected by malaria in 13 SSA countries. Malaria infection remains one of the main killers of children aged 6–59 months in the SSA. This study revealed that older under-five children living in large families with low incomes in rural areas are most vulnerable to malaria infection. Our results clearly indicate that ITN utilization and improved housing are promising means to effectively prevent malaria infection among children aged 6–59 months. It is therefore important to note that households with low wealth quintiles and rural residents should be prioritized in any mass distribution of ITNs. This has to be accompanied by education using mass media to enhance community awareness.

## Background

Although malaria is a preventable and curable disease, it remains a major public health problem, particularly in low and middle-income countries [1–3]. In 2020, nearly half of the world’s population was at risk of malaria. About 241 million people were infected and over 627 000 died, indicating 14 million more cases and 69 000 more deaths in 2020 compared to 2019 [4,5]. The African region, which carries a disproportionately high share of the global malaria burden, accounted for 95% of malaria cases and 96% of malaria deaths in the same year [6]. The risk was even considerably higher among infants, under-five children, pregnant women, and patients with HIV/AIDS [7–10]. In 2020, under-five children accounted for about 80% of all malaria deaths in the World Health Organization (WHO) African region [6].

The control and eradication of malaria demand a multifaceted approach [11]. Since the early 20^th^ century, the world has put effort into organizing different programs and campaigns to control and eradicate malaria and save millions of lives. These include the Malaria Commission of the League of Nations (1920), the global eradication campaigns (1950), the Ministerial Conference on Malaria (1992), the Roll Back Malaria (RBM) movement (1998), the Global Fund (2015), Millennium Development Goals (MDGs) and Sustainable Development Goals (SDGs) [12–15]. Despite substantial progress has been made over the past decades, the prevalence is unacceptably high, especially in some African countries [16,17]. In 2020, four Sub-Saharan African (SSA) nations, including Nigeria (31.9%), Dr. Congo (13.2%), Tanzania (4.1%), and Mozambique (3.8%), accounted for over half (53%) of all malaria deaths worldwide [6].

Every 75 seconds, a child under five dies of malaria [18]. Mainly, children aged between six months and five years are at the highest risk for malaria because during this period they have lost maternal immunity and did not yet developed specific immunity to the infection [19,20]. Under-five children bear the highest burden of malaria in SSA [21,22]. Many countries in the region have missed the MDGs and are lagging far behind the SDGs’ targets of reducing malaria case incidence and mortality rates by at least 90% by 2030 [23,24]. Although there are many factors responsible for this, poverty takes the lion’s share [25]. Malaria is commonly recognized as a disease of poverty which explains why its incidence is concentrated in the world’s poorest countries [26–28]. In addition to poverty, previous studies have shown that place of residence, insecticide-treated bed net (ITN) utilization, housing conditions (floor, wall, and roof materials), children’s anemia level, women’s education, and media access have the highest impact on malaria burden among those under-five children [29–33].

Despite malaria among under-five children being well recognized as a common public health problem, its prevalence and different determinants have been less investigated in SSA. The persistent and overwhelming burden of deaths among under-five children indicates the urgent need for collective action against malaria. Adequate understanding of the socio-economic, environmental, and cultural factors is important to successfully prevent the burden. Besides if the SDG-3 targets are to be met, coordinated action is required toward not only sustaining current rates of decline but also accelerating progress. Therefore, the objective of this study was to assess the prevalence of malaria among children aged 6–59 months and the potential factors associated with the malaria risk in 13 SSA countries. Our study provided evidence-based recommendations to control and eliminate malaria in those children on a large scale in SSA.

## Materials and methods

### Study design, setting, and period

This study was conducted on a secondary data from the recent Malaria Indicator Surveys (MIS) of 13 SSA countries. These countries are Burkina Faso, Ghana, Guinea, Kenya, Liberia, Madagascar, Mali, Malawi, Mozambique, Nigeria, Sierra Leone, Senegal, and Tanzania (**Table 1**).

**Table 1:** Country’s malaria indicator year of survey.

Country	Year of survey	Country	Year of survey
Burkina Faso	2017/18	Malawi	2017
Ghana	2019	Mozambique	2018
Guinea	2021	Nigeria	2015
Kenya	2020	Sierra Leone	2016
Liberia	2016	Senegal	2020/21
Madagascar	2016	Tanzania	2017
Mali	2021		

### Sources of data and sampling procedure

This study used the most recent MIS data from 13 SSA countries conducted from 2015 to 2021. MIS is a cross-sectional nationally representative survey undertaken across Low- and Middle-Income Countries (LMICs), developed by the Monitoring and Evaluation Working Group (MERG) of RBM. MIS collects national and regional or provincial data from a representative sample of respondents. It measures key indicators that are considered important which enables countries to generate data useful to inform policy makers and improve malaria control programs. All MIS use the same standardized data collection procedures, sampling, questionnaires, and coding, making the results comparable across countries.

To assure national representativeness, the survey was based on a two-stage sampling technique. In the first stage, the selection of proportional clusters/enumeration areas was performed using each country’s most recent population and housing census as a sampling frame. In the second stage, households were sampled using systematic random sampling from the newly created household listing selection of census enumeration areas. A detailed description of the MIS sampling design and data collection procedures can been found in each country’s DHS report. A total of 74,976 parents/guardians were interviewed in 13 SSA countries. For this study, a total of 64,247 children aged 6–59 months, who had slept last night and households selected for hemoglobin were included. We weighted the sample using the individual weight of women (hv005) to produce the proper representation. Hence, sample weights were generated by dividing (hv005) by 1,000,000 and the total weighted sample of 60,541 children was used for the analysis.

### Variables of the study

#### Outcome variable

The outcome variable was malaria infection detected by a rapid diagnostic test or microscopy among children aged 6–59 months.

#### Independent variables

Based on previous literature, theoretical and practical significance, the independent variables for this study were the age of the child, sex of the child, mother’s educational level, age of household head, wealth index, number of under-five children, the household has radio, the household has television, the household has electricity, source of drinking water, type of toilet facility, main floor material, main wall material, main roof material, type of bed net, number of mosquito net, anemia level, residence, community ITN usage, community poverty, community media usage, and community-women education (**Table 2**).

**Table 2:** Independent variables of malaria among children aged 6–59 months in SSA.

Variables	Categorization/operationalization
**Individual level variables**	
Age of child	The age of the child was categorized as 6–12, 13–23, 24–35, 36–47, and 48–59 months
Sex of child	The sex of the child was categorized as male or female.
Mothers educational level	The mother’s educational status was categorized as uneducated, primary, secondary, and above
Age of household head	The age of household heads was categorized as 13–24, 25–34, 35–49, and ≥50 years.
Wealth index	The wealth index was categorized as poorest, poorer, middle, richer, and richest.
Number of under-five children	The number of under-five children was categorized as one, two, three & more
Household has radio	Household radio access was categorized as No and Yes
Household has television	Household television access was categorized as No and Yes
Household has electricity	Household electricity access was categorized as No and Yes
Source of drinking water	The source of drinking water was categorized as Unimproved and Improved
Type of toilet facility	The type of toilet facility was categorized as Unimproved and Improved
Main floor material	The main floor material was categorized as Unimproved and Improved
Main wall material	The main Wall material was categorized as Unimproved and Improved
Main roof material	The main roof material was categorized as Unimproved and Improved
Type of bed net	The type of bed net was categorized as Untreated and Treated
Number of mosquito net	The number of mosquito nets was categorized as none, one, two, three & more
Anemia level	The anemia level of children was categorized as severe, moderate, mild, and not anemic
**Community level variables**	
Residence	The type of place resident was grouped as urban and rural
Community ITN usage	Community-level ITN usage was categorized as low and high. “Low” refers to communities in which < 50% of respondents use ITN while “high” indicates communities in which ≥ 50% of respondents use ITN.
Community poverty	Community-level poverty was categorized as low if the proportion of low wealth quintile (poorest and poorer) households was <50% and high if the proportion was ≥ 50%.
Community media usage	Community media usage was categorized as low if communities in which < 50% of respondents had media exposure and high if ≥ 50% of respondents had media exposure.
Community-women education	Community women’s education was categorized as low if communities in which < 50% of respondents had primary and above education and high if ≥ 50% of respondents had attended primary and above.

### Data processing and analyses

Thirteen SSA countries datasets were appended together to explore the pooled prevalence of malaria and its associated factors among children aged 6–59 months. STATA version 14.2 was used for data cleaning, coding, and statistical analysis. Frequencies and percentages were used to describe the background characteristics of the study participants. To identify associated factors of malaria we used the multilevel logistic regression since MIS data are hierarchical in nature Four models were fitted which comprised the null model (model 0) without any explanatory variables, Model I with individual-level variables only, Model II with community-level factors only, and Model III with both individual-level and community-level variables.

The Intra-class Correlation Coefficient (ICC), and the Median Odds Ratio (MOR) were computed to assess the clustering effect/variability. The ICC was calculated for each of the models using the formula, ICC = the variance of each model/ (variance of each model + 3.29), and the MOR was calculated as; MOR = exp(0.95√ cluster level variance). Proportional Change in Variance (PCV) was computed for models I, II, and III with respect to the variance in the empty model as PCV = (variance of the empty model—variance of the model with more terms (model I, II, or III) / variance of the empty model, to show the power of the factors in the model to explain malaria prevalence. All variables with a p-value ≤0.2 in the bi-variable analysis were fitted in the multivariable model. Adjusted OR (AOR) with 95% CI and p<0.05 were presented to reveal significantly associated factors.

#### Ethics approval and consent to participate

Permission to access the data was obtained from the measure DHS program (http://www.dhsprogram.com) via online request. The website and the data used were publicly available with no personal identifier. All methods were carried out in accordance with relevant guidelines and regulations.

## Results

### Background characteristics of study participants

A total of 60,541 under-five children from 13 SAA countries were included in this study. Of the total, 46.72% were in the age group of 36–59 months. About 38.13% and 44.80% of the mothers respectively did not attended formal education and were categorized in low-wealth quintiles (poorer and poorest). Regarding media access, 33,307 (55.03%) of the households listed radio and 17,941 (29.64%) watched television at least once a week. Nearly three-fourths (74.03%) of study participants were rural residents, and in total 33,972 (56.11%) uses treated mosquito nets. The majority of households had unimproved toilet facilities, floor, wall, and roof materials (**Table 3**).

**Table 3:** Socio-demographic characteristics of study participants in Sub-Saharan Africa.

Variables	Categories	Weighted frequency (%)
Age of child (months)	6–12	7,475 (12.35)
	13–23	11,616 (19.19)
	24–35	13,169 (21.75)
	36–47	13,983 (23.10)
	48–59	14,298 (23.62)
Sex of child	Male	30,775 (50.83)
	Female	29,766 (49.17)
Mothers educational level	No education	23,084 (38.13)
	Primary	13,492 (22.29)
	Secondary and above	23,965 (39.59)
Age of household head	13–24	2,861 (4.72)
	25–34	14,531 (24.00)
	35–49	23,599 (38.98)
	≥50	19,549 (32.29)
Wealth index	Poorest	13,815 (22.82)
	Poorer	13,307 (21.98)
	Middle	12,107 (20.00)
	Richer	11,520 (19.03)
	Richest	9,792 (16.17)
Number of under-five children	One	18,269 (30.18)
	Two	21,742 (35.91)
	Three & more	20,529 (33.91)
Household has radio	No	27,221 (44.97)
	Yes	33,307 (55.03)
Household has television	No	42,585 (70.36)
	Yes	17,941 (29.64)
Household has electricity	No	43,599 (72.02)
	Yes	16,941 (27.98)
Source of drinking water	Unimproved	20,682 (34.16)
	Improved	39,859 (65.84)
Type of toilet facility	Unimproved	33,732 (55.72)
	Improved	26,809 (44.28)
Main floor material	Unimproved	33,731 (55.72)
	Improved	26,810 (44.28)
Main wall material	Unimproved	32,479 (53.65)
	Improved	28,062 (46.35)
Main roof material	Unimproved	35,179 (58.11)
	Improved	25,362 (41.89)
Type of bed net	Untreated	26,569 (43.89)
	Treated	33,972 (56.11)
Number of mosquito net	None	11,977 (19.78)
	One	11,098 (18.33)
	Two	14,268 (23.57)
	Three & more	23,198 (38.32)
Anemia level	Sever	1,826 (3.02)
	Moderate	22,883 (37.81)
	Mild	15,753 (26.03)
	Not anemic	20,064 (33.15)
Residence	Urban	15,722 (25.97)
	Rural	44,819 (74.03)
Community ITN usage	Low	30,751 (50.79)
	High	29,790 (49.21)
Community poverty	Low	30,933 (51.09)
	High	29,608 (48.91)
Community media usage	Low	29,746 (49.13)
	High	30,795 (50.87)
Community-women education	Low	30,818 (50.90)
	High	29,723 (49.10)

### Prevalence of malaria

**Fig 1:**
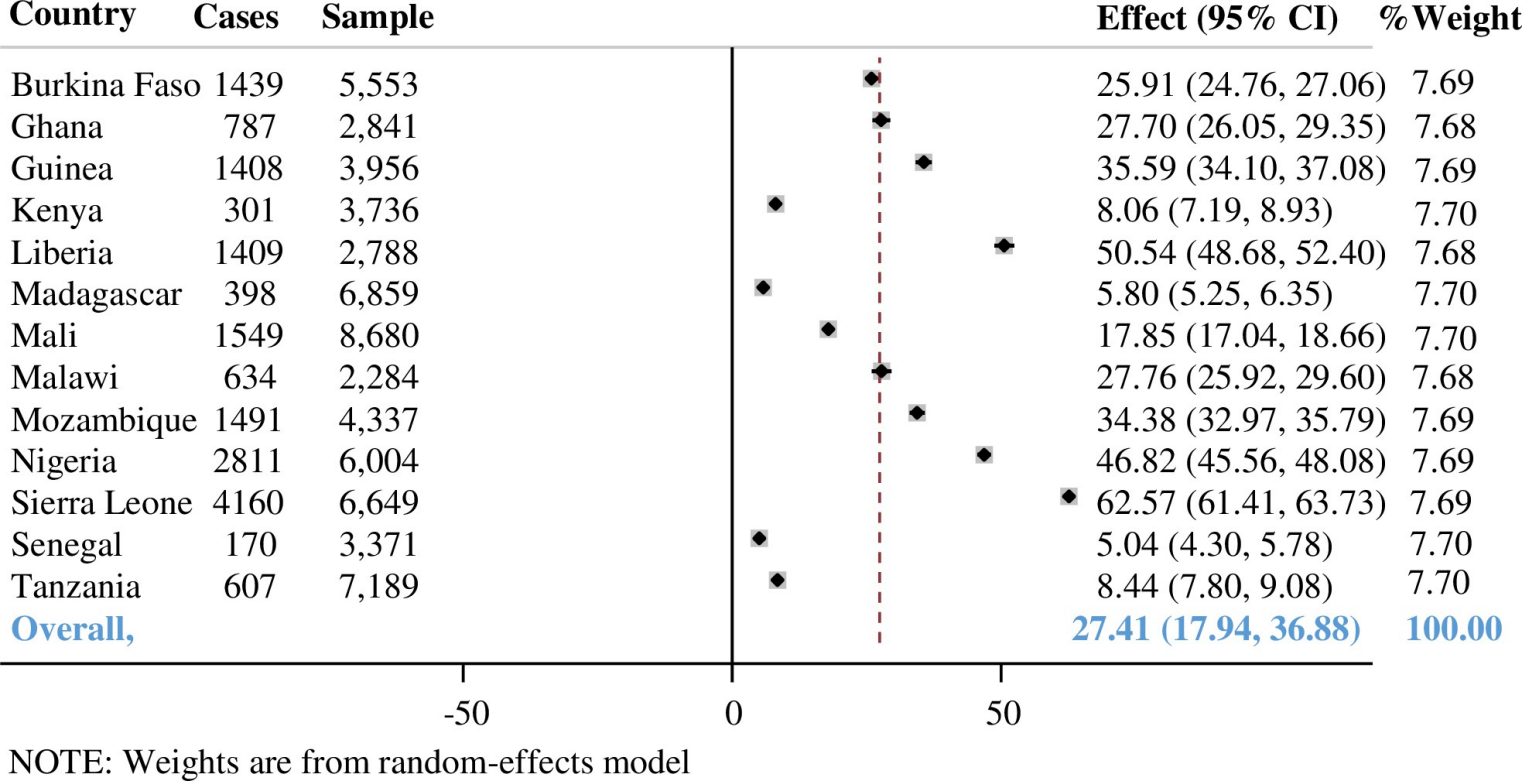
Pooled prevalence of malaria in under-five children in Sub-Saharan Africa.

With inter-country variations, the overall pooled prevalence of malaria among children aged 6–59 months in SSA was found to be 27.41% (95% CI: 17.94%, 36.88%), ranging from 5.04% in Senegal to 62.57% in Sierra Leone (**Fig 1**).

### Multilevel logistic regression analysis

#### Model comparison and random effect analysis

The result of random effect analysis depicts that the ICC of the null model was 0.19, indicating that 19% of the total variability in malaria prevalence was attributable to between-cluster variability, while about 81% was due to individual differences. The null model MOR was 2.24, which indicates that a child from a cluster with high malaria prevalence has a 2.24 times higher probability of being infected than a child from a cluster with lower malaria prevalence. Model III was the best-fitted model since it has the highest log likelihood (-17737) and the lowest deviance (35474) value. The PCV of model III was 72%, meaning that about 72% of the total variability in the malaria prevalence was explained by the full model (**Table 4**).

**Table 4:** Multivariable multilevel logistic regression analysis results of both individual-level and community-level factors associated with malaria in Sub-Saharan Africa.

Variables	Categories	Null model	Model I (AOR 95% CI)	Model II (AOR 95% CI)	Model III (AOR 95% CI)
Age of child (months)	6–12		1.00	—	1.00
	13–23		1.62 (1.47–1.80)***	—	1.63 (1.47–1.81)***
	24–35		2.44 (2.21–2.70)***	—	2.44 (2.21–2.70) ***
	36–47		3.54 (3.21–3.91)***	—	3.54 (3.21–3.91) ***
	48–59		4.33 (3.92–4.78)***	—	4.32 (3.91–4.77) ***
Sex of child	Male		1.00	—	1.00
	Female		0.99 (0.94–1.05)	—	0.99 (0.95–1.05)
Mothers educational level	No education		1.00	—	1.00
	Primary education		0.77 (0.72–0.83)**	—	0.78 (0.73–0.84)**
	Secondary & above		0.98 (0.95–1.08)	—	0.99 (0.97–1.11)
Age of household head	13–24		1.00	—	1.00
	25–34		0.98 (0.86–1.13)	—	0.98 (0.86–1.13)
	35–49		1.03 (0.90–1.18)	—	1.03 (0.90–1.18)
	≥50		1.10 (0.95–1.26)	—	1.10 (0.95–1.26)
Wealth index	Poorest		1.00	—	1.00
	Poorer		0.81 (0.75–0.89)***	—	0.83 (0.77–0.90)***
	Middle		0.57 (0.52–0.61)***	—	0.57 (0.52–0.62)***
	Richer		0.33 (0.30–0.38)***	—	0.35 (0.32–0.39)***
	Richest		0.15 (0.13–0.18)***	—	0.16 (0.14–0.19)***
Number of under-five children	One		1.00	—	1.00
	Two		1.19 (1.12–1.27)**	—	1.20 (1.12–1.28)***
	Three & more		1.34 (1.25–1.45)***	—	1.35 (1.26–1.45)***
Household has radio	No		1.00	—	1.00
	Yes		1.05 (0.99–1.11)	—	1.01 (0.98–1.09)
Household has television	No		1.00	—	1.00
	Yes		0.85 (0.77–0.93)**	—	0.86 (0.78–0.95)**
Household has electricity	No		1.00	—	1.00
	Yes		0.67 (0.61–0.73)***	—	0.68 (0.62–0.75)***
Source of drinking water	Unimproved		1.00	—	1.00
	Improved		0.98 (0.93–1.04)	—	0.97 (0.92–1.04)
Type of toilet facility	Unimproved		1.00	—	1.00
	Improved		0.98 (0.92–1.04)	—	1.00 (0.93–1.06)
Main floor material	Unimproved		1.00	—	1.00
	Improved		0.64 (0.56–0.72)***	—	0.65 (0.57–0.73)***
Main wall material	Unimproved		1.00	—	1.00
	Improved		0.68 (0.58–0.78)**	—	0.73 (0.64–0.84)***
Main roof material	Unimproved		1.00	—	1.00
	Improved		0.78 (0.65–0.92)***	—	0.70 (0.51–0.93)***
Number of mosquito net	None		1.00	—	1.00
	One		1.08 (0.99–1.18)	—	1.07 (0.98–1.17)
	Two		0.90 (0.82–0.98)*	—	0.89 (0.82–0.98)**
	Three & more		0.93 (0.85–1.01)	—	0.91 (0.83–0.99)**
Type of bed net	Untreated		1.00	—	1.00
	Treated		0.55 (0.50–0.62)***	—	0.56 (0.51–0.62)***
Anemia level	Sever		1.00	—	1.00
	Moderate		0.27 (0.23–0.32)***	—	0.27 (0.23–0.32)***
	Mild		0.10 (0.09–0.13)***	—	0.11 (0.09–0.13)***
	Not anemic		0.06 (0.04–0.07)***	—	0.05 (0.04–0.06)***
**Community-level variables**					
Residence	Urban		—	1.00	1.00
	Rural		—	2.35 (2.19–2.52)***	2.16 (2.06–2.27)***
Com. ITN usage	Low		—	1.00	1.00
	High		—	0.28 (0.14–0.39)*	0.40 (0.24–0.63)***
Com. poverty	Low		—	1.00	1.00
	High		—	2.85 (2.66–3.07)**	2.66 (2.53–2.84)***
Com. media usage	Low		—	1.00	1.00
	High		—	0.97 (0.83–1.15)	0.94 (0.81–1.11)
Com. women’s education	Low		—	1.00	1.00
	High		—	0.85 (0.78–0.90)***	0.81 (0.73–0.88)***
**Random effect**					
	Variance	0.75	0.42	0.29	0.21
	ICC	0.19	0.11	0.08	0.06
	MOR	2.24	1.67	1.39	1.18
	PCV	Reff	44.00	61.33	72.00
**Model comparison**					
	Log likelihood ratio	-21258	-18788	-20887	-17737
	Deviance	42516	37576	41774	35474

ICC = Inter cluster correlation coefficient, MOR = Median odds ratio, PCV = proportional change in variance. AOR = adjusted odds ratio; CI = confidence interval, Com. women’s education = community women’s education; Com. ITN usage = community Insecticide-treated bed nets; Com. Media = community media usage; Com. Poverty = community poverty status.

* = P-value < 0.05

** = P-value < 0.01

*** = P-value < 0.001.

#### Fixed effect analysis

Based on the best model (Model III) multilevel logistics regression analysis, both individual and community-level variables were significant determinants of malaria in under-five children. Among individual-level variables; Children aged 13–23, 24–35, 36–47, and 48–59 months, maternal primary education, wealth index, the number of under-five children, the household has television, the household has electricity, main floor materials, main wall materials, main roof materials, anemia level, type of bed net and the number of mosquito nets were significantly associated variables with malaria. Additionally, community-level variables including place of residence, community ITN utilization, community poverty, and community women’s education were significant factors for malaria (**Table 4**).

## Discussion

So far, the WHO-recommended malaria prevention strategies, such as vector control and chemoprophylaxis, have played a major role in reducing the burden in SSA countries [34,35]. However, complete elimination is jeopardized by different factors [36,37]. Pandemic, poverty, biological determinants (affected population, parasites, and vectors), climatic and environmental factors, weak health care systems, and political unrest are among the obstacles [38,39]. For example, in 2020, the malaria mortality rate increased for all ages due to service disruptions by the COVID-19 pandemic [40]. Additionally, a lack of political commitment and limited international and domestic funding for malaria elimination will slow the fight against the disease [41,42].

One of the WHOs strategic frameworks is to transform malaria surveillance into a core intervention. This includes identifying the most affected population, detecting possible determinants, providing a targeted response, and assessing the effectiveness of malaria protective tools [43]. Therefore, systematically investigating and identifying the potential factors of malaria among children aged 6–59 months will support the WHOs strategies for reducing malaria burden in many ways. Our study confirmed that the prevalence of malaria infection among children aged 6–59 months in the thirteen SSA countries was found at 27.41%. Senegal has the lowest (5.04%) prevalence while Sierra Leone bears the highest (62.57%). The variation among countries could be attributed to differences in socio-economic status, geographic situations, seasonal changes, health care system strength, national policy, and political commitments [44,45]. This study also revealed that both individual and community-level factors have a significant impact on malaria infection in those children.

The results showed a parallel correlation between malaria infection and child age. The odds of infection increase as the age of the child increases. Older under-five children were more likely to be infected as compared to younger ones [46–48]. Passively transferred maternal antibodies, such as lactoferrin and secretory IgA, provide protection to infants against malaria [49,50]. Besides, older children are more exposed to mosquitoes compared to younger ones, being more protected by their parents. The other possible reason why the infection rate is higher in older children is the fact that the insect prefers to bite older children, not infants [51]. Parents of older under-five children should be well informed by health workers via different communication tools, that their children are a group at high risk for malaria. Early information conveyed by health workers to parents enhancing knowledge and awareness about the risk of malaria for their children and efforts to prevent it.

Under-five children from whom the mother attended primary education were lower risk of becoming infected by malaria. Similar results were found on community-level, i.e. the risk of malaria infection was low among children originating from a community with high women’s education [46,52]. Sub-Saharan Africa is a vast region where women’s education is relatively low due to cultural, geographical, and socio-economic conditions [53]. Educated mothers usually have a better standard of living and better comprehension of malaria-related information that could help them to protect their children [54]. Mothers expand their knowledge about the impact of mosquito nets utilization, indoor residual spray, and other potential preventive measures for malaria [55,56]. In addition, it is crucial that the mother is aware of the signs and symptoms of malaria infection in order to get timely and appropriate treatment for their children [57].

Our study revealed an inverse relationship between the household wealth quintiles and malaria infection. As the household income increases, the odds of malaria infection decrease [58–60]. It was found that malaria infection was higher among children who are from a community with high poverty. The risk of being infected by malaria among children from the richest household was 84% less compared to the poorest. Malaria is often referred to as the epidemic of poverty [26]. In this study, almost a quarter of children are from the poorest household. A plausible explanation is that malaria bankrupts households and national economies, lowers worker productivity, and discourages investment [61]. In essence, less Malaria leads to less poverty, leading to fewer expenses for households and improving their income. This also could lead to malnutrition among those children [21,62]. In our study, anemia status was a strong determinant of malaria infection. Poor nutritional status is associated with vulnerability to progression from malaria infection to disease [63].

Likewise, the number of under-five children in a household was found to be a significant determinant of malaria. The chance of infection increases as the number of children in the household increases. A previous study [64] revealed that one additional member in the household increases the likelihood of malaria infection by about 6.5%. Large family size mostly creates a congested space to sleep in the house and a shortage of mosquito nets [65]. The WHO recommends ITN utilization as a vital component of malaria control and elimination strategies as it is highly effective in preventing infection and reducing disease transmission [66]. Therefore, wide access to ITN for the household and the community is of crucial importance to limit the rate of malaria infection [67,68]. Our study confirmed that the infection is less likely in a household where an abundant number of mosquito nets is available. The risk is even much lower in children from a household with a mosquito net treated by insecticide and in a community with high ITN utilization.

Housing conditions including the possession of a television, electricity, and improved construction materials (floor, wall, and roof materials) have reduced the odds of malaria infection among children. Mass media (television, radio, newspaper) plays an important role to prevent malaria by disseminating information on the utilization of ITN and improving the housing condition [69]. The quality of the house or material used for construction affect the entry of mosquitoes into dwelling places [47,70]. Researchers revealed that modern and well-built housing played a vital role in controlling human exposure to mosquitoes [71,72]. Particularly in rural residents, the housing condition deteriorated which created a suitable environment for vector-borne diseases like malaria [73]. Our study confirmed that children residing in rural areas were 2.16 times more likely to be infected by the malaria parasite. Additionally, rural residents consistently face physical and financial problems to access health services for malaria treatment, resulting in high malaria infection prevalence.

Our study has some strengths and limitations. It used large nationally representative samples with appropriate statistical modeling. The use of large nationally representative data and multilevel analysis helps to provide more robust estimates of observed associations as well as enhance the generalizability of the results. Despite the strengths, this study used cross-sectional data, which did not indicated a temporal relationship between the factors and malaria infection. No important covariates, such as indoor residual spraying, vaccination, and other comorbid conditions, were incorporated in the study as the MIS did not provide a complete record of these variables.

## Conclusions and recommendations

Almost 3 out of 10 children were infected by malaria in the thirteen Sub-Saharan countries. Malaria infection remains one of the main killers of children aged 6–59 months in the SSA. This study revealed that older under-five children living in large families with low incomes in rural areas are most vulnerable to malaria infection. It also presented that ITN utilization and improved housing are promising means to effectively prevent malaria infection among children aged 6–59 months. It is therefore important to note that households with low wealth quintiles and rural residents should be prioritized in any mass distribution of ITNs. This has to be accompanied by education using mass media to enhance community awareness.

## Abbreviations

AOR: Adjusted Odds Ratio
DHSs: Demographic and Health Surveys
ICC: Intra cluster correlation coefficient
MIS: Malaria Indicator Survey
MOR: median odds ratio
PCV: a proportional change in variance
SSA: Sub-Saharan Africa
WHO: World Health Organization
